# ASO Author Reflections: Pancreatic Neuroendocrine Tumor Recurrence and Survival Predicted by Ki67

**DOI:** 10.1245/s10434-018-7019-z

**Published:** 2018-12-05

**Authors:** Els J. M. Nieveen van Dijkum

**Affiliations:** Department of Surgery, Cancer Center Amsterdam, Amsterdam UMC, University of Amsterdam, Amsterdam, The Netherlands

## Past

Risk classification of pancreatic neuroendocrine tumors is infrequently used for treatment or follow-up strategies. Multiple reasons for this lack of use can be identified. First, risk classifications are based on small retrospective data or large nationwide cohorts, with either low statistical power or high risk of bias. Second, patients are not always referred to centers of excellence, where treatment options are possibly better weighed and risk classifications known and incorporated in decisions regarding diagnostics or treatment. Referral to centers of excellence increases volume in these centers, further improving patient outcomes.^1^ A final third reason for the lack of use of risk classifications is the indolent behavior of pancreatic neuroendocrine tumors. Ten-year survival rates over 75%, as well as low recurrence rates, do not facilitate prospective trials on diagnostic or treatment strategies. However, if increasing data are suggestive of certain risk factors associated with tumor recurrence, collaborating centers must evaluate again and again if prospective studies are to be initiated.

## Present

Our study^2^ consisted of 240 patients from three centers of excellence who had undergone curative resections for well-differentiated neuroendocrine pancreatic tumors. Patients with a Ki67 score of ≤ 5% had a threefold lower chance of developing recurrent disease compared with patients with a Ki67 score of 6–20%, i.e. 14% and 41%, respectively. Time to recurrence was 34 months in the Ki67 ≤ 5% group versus 16 months in the Ki67 6–20% group. Survival after recurrence was similar in both groups, at 44.9 months. As the World Health Organization (WHO) divides patients into grade 1 (Ki67 ≤ 3%) and grade 2 (Ki67 3–20%), a comparison was made between the WHO classification and our grading system. Our classification was 10% better than the WHO classification because patients with a Ki67 score of between 3 and 5% had a low risk of recurrence and should not be graded as WHO grade 2.

The 5-year recurrence-free survival and 10-year disease-specific survival periods were 90 and 91%, respectively, for Ki67 0–5%, and 55 and 26%, respectively, for Ki67 6–20% (*p* < 0.001). These data are probably not completely unexpected, however they will enable more personalized strategies for patients. Therefore, different follow-up schedules are suggested for low- versus high-risk patients.

## Future

Recurrence after curative resection of pancreatic neuroendocrine tumors should be prevented in the future. Different treatment options are available for these patients, such as chemotherapy, peptide receptor radionuclide therapy (PRRT) or tyrosine kinase inhibitors, but first the decision has to be made as to whether the available risk classification is solid enough to select patients for adjuvant treatment.^3^^–^6 In addition, it remains unknown if treatment of recurrence has a poorer outcome than adjuvant treatment focusing on the prevention of recurrences. This issue can only be solved with a prospective study, possibly a trial or pilot study with adjuvant treatment, or a wait and see approach. If the patients with a 41% recurrence risk (Ki67 6–20% patients) are included in all centers of excellence, we would probably be able to answer that question within 3 years, given that recurrences were detected after a mean of 16 months. Still, the incidence of neuroendocrine pancreatic tumors defies the possibility of prospective trials with strong evidence for treatment effects. In a perfect world, all patients would be directed to a limited number of centers of excellence per country, enabling the best outcomes.
